# Editorial for Special Issue “Cellular Senescence: Recent Cellular Advances and Discoveries”

**DOI:** 10.3390/biomedicines12122796

**Published:** 2024-12-09

**Authors:** Karen Carmelina Crasta, Francesca Faggioli

**Affiliations:** 1 Healthy Longevity Translational Research Programme, Yong Loo Lin School of Medicine, National University of Singapore, Singapore 117456, Singapore; 2 Department of Physiology, Yong Loo Lin School of Medicine, National University of Singapore, Singapore 117593, Singapore; 3 Centre for Healthy Longevity, National University Health System, Singapore 117456, Singapore; 4 NUS Centre for Cancer Research, Yong Loo Lin School of Medicine, National University of Singapore, Singapore 117599, Singapore; 5 IRCCS Humanitas Research Hospital, Rozzano, 220089 Milan, Italy; 6 Institute of Genetics and Biomedical Research, UoS of Milan, National Research Council, 20132 Milan, Italy

Cellular senescence has emerged as a fascinating frontier in biological research and now presents profound implications across diverse fields, from aging research to cancer therapy. Recent advancements have reshaped our understanding of senescence, revealing its intricate role in both health and disease.

At its core, cellular senescence represents a state where cells cease to divide and instead enter a state of stable growth arrest. Initially recognized as a safeguard against cancer, this process prevents damaged cells from proliferating uncontrollably, preserving tissue integrity and acting as a barrier against cancer. One of the most striking revelations is the dual nature of senescence. Indeed, the accumulation of senescent cells in tissues over time has been linked to inflammation, tissue dysfunction, and the overall degenerative processes observed in aging. Recent studies have also illuminated its dynamic features, demonstrating that senescent cells actively communicate with their environment through its secretome, the senescence-associated secretory phenotype (SASP) [1]. The SASP can influence neighboring cells and tissues to either promote inflammation and tissue deterioration or contribute to tissue repair and regeneration under certain conditions. However, the story of senescence does not end here; it unfolds into a multifaceted narrative enriched by recent discoveries. This Special Issue aims to capture some of these recent advances from various research areas.

Long non-coding RNAs (lncRNAs) have emerged as important modulators in senescence and senescence-associated etiologies. For instance, the lncRNA MIR31HG was shown to regulate the expression and secretion of a SASP subset [2]. In this Special Issue, Stefano Bellosta and colleagues identified significant upregulation of specific lncRNAs regulating PURPL and NEAT1 and increased RRAD mRNA levels in replicatively senescent human aortic vascular smooth muscle cells, associated with cardiovascular disease and atherosclerosis [3]. The modulation of RRAD, PURPL, and NEAT1 levels thus could serve as a potential marker for studying anti-aging therapies.

Yet another burgeoning area is the role of ion channels, where alterations in ion channel activity, expression, and regulation may influence the onset and progression of senescence. Changes in calcium (Ca^2+^) signaling have been implicated in triggering senescence, while chloride channels have been linked to cell cycle arrest and the regulation of cellular pH [4]. These findings highlight the critical role of ion channel dynamics in both initiating and maintaining the senescent state. Pidder Jansen-Durr, Maria Cavinato and colleagues shed light on the role of CLCA2, a calcium-activated chloride channel accessory protein, in cellular senescence and skin aging. CLCA2 was found to be upregulated in senescence pathways, while its depletion accelerated senescence. In 3D skin models, CLCA2 knockdown in fibroblasts resulted in skin features resembling aged skin, highlighting its role in maintaining skin homeostasis. Their findings suggest that CLCA2 is a key regulator of senescence and could be targeted in strategies to address skin aging.

Senescent cells are also known to display altered metabolic states [5]. For instance, recent studies have shown that specific lipid species plays a critical role in senescence [6], contributing, for instance, to the low-grade inflammation associated with SASP [7]. Interestingly, recent findings showed multiple SASP markers that were elevated in rapid agers, and not in healthy agers, were also associated with specific metabolites such as specific lipids, amino acids and xenobiotics [8]. Here, Rosalinde Masereeuw and colleagues illustrate a case in point for toxic metabolites in SASP-associated pathologies [9]. The authors describe the mechanism underlying how accumulation of protein-bound uremic toxins, normally eliminated via the urine in healthy kidneys, promote SASP-triggered inflammation. This eventually results in the development of kidney fibrosis and chronic kidney disease, pointing to the importance of a deeper understanding of the associated mechanisms.

Several metabolic pathways result in the generation of mitochondrial reactive oxygen species (ROS), thought of as a tightly regulated signaling process that induces cell senescence and aging. Indeed, the mitochondrial-targeted catalase (mCAT), which reduces mitochondrial ROS, has been suggested to have lifespan-extending benefits [10]. Despite this, important groundbreaking work by Chris Wiley and colleagues now indicates that mCAT did not reduce senescence markers or the SASP in cultured cells or in aged mice [11]. This suggests that mitochondrial ROS may not be a primary driver of senescence during natural aging, highlighting the complexity of its role in the aging process.

Extracellular vesicles (EVs) have emerged as important yet underappreciated cellular components of the senescent secretome, functioning as intercellular communicators [12,13]. Aging can potentially impact the cargo of circulating EVs. Remarkably, administration of exosomes derived from young mice was shown to alter the expression pattern of aging-associated molecules in aged mice, emphasizing the critical role of EVs [14]. Here, using aged animals, Ionara Siquera and colleagues reported EV-derived microRNA and microarray data associated with age-related oxidative stress that can potentially drive cardiovascular aging and cardiac disease [15]. On the other hand, EVs could also play an anti-senescence role. In elegant work conducted by Gerardo Ferbeyre and colleagues, increased secretion of EVs and cytokines by senescent macrophages allowed senescent murine embryonic fibroblasts to re-enter the cell cycle, pointing to an underlying mechanism for macrophage-associated pro-tumorigenic activity. This propounds the use of senolytics - compounds designed to selectively eliminate senescent cells- to eliminate macrophages as anti-cancer therapy.

Along the lines of tumorigenic roles of senescence, recent studies have emphasized its role in chronic inflammatory liver disease resulting in liver cancers. In addition, senescence triggers a stem-like state that can lead to drug-resistant aggressive tumor clones. Here, Matteo Donadon and colleagues [16] thoroughly evaluate the contribution of senescence to primary and metastatic liver cancers and its role in therapy resistance, highlighting challenges that could be encountered when embarking on the use of senescence as a weapon to target liver cancers.

Indeed, the therapeutic potential of targeting senescence has captured significant attention. Researchers are exploring senolytic drugsas a means to alleviate age-related conditions and possibly extend health span [17]. These have shown impact at the fundamental level; for instance, selective elimination of radiation-induced senescent cells with micronuclei by ABT-263 was found to alleviate micronuclei-related STING activation [18,19]. Initial studies in animal models have shown promising results, spurring cautious optimism that such interventions could translate into clinical benefits for humans. Here, Zuzarte et al. focus on aromatic plants, particularly their bioactive volatile compounds, such as monoterpenes, which have shown potential anti-aging effects [20]. There has been a lack of comprehensive documentation that examines the mechanisms behind these effects and the specific molecular interactions involved. Their review systematically illuminates current knowledge on how volatile monoterpenes affect the aging process to identify the mechanisms of action and to establish possible relationships between the chemical structure of these compounds and their biological effects. Since most of the therapeutic agents currently in development to combat aging-related pathologies target senescent cells, it will be interesting to explore plant monoterpenes as senolytics.

Lastly, Guang Yao offers a unique perspective on quiescence-origin senescence [21]. This opinion paper introduces a new concept called “quiescence-origin senescence,” where cells can directly transition from a dormant (quiescent) state into senescence, bypassing the typical process of cell proliferation. He proposes a continuum between quiescence and senescence, with deepening quiescence acting as a precursor to senescence. It explores the triggers and regulatory mechanisms of this transition, highlighting its biological significance. Since most cells in the human body are in a quiescent state rather than proliferating, understanding this process could lead to new strategies for treating age-related diseases and improving tissue regeneration. This could also have implications for senescence escape, another emerging concept particularly important for therapy-induced senescence and drug resistance [22].

Another promising future endeavor that warrants being pursued is the interplay between cellular senescence and circadian regulation. Previous evidence has implicated disruptions of the circadian clock to aging and aging-related disease such as cancer [23,24]. The circadian clock regulates the expression and activity of biological processes implicated in aging, such as mitochondrial metabolic and antioxidant enzymes. Hence, defining the molecular circuitry that connects the circadian rhythm, cell cycle regulation and senescence and its impact on physiological processes is a promising future endeavor that warrants being pursued. This could also be useful for space exploration as space flight has been postulated to contribute to frailty [25,26], and circadian dysregulation has been proposed as a key factor in aging both on Earth and in space [26].

As we navigate the complexities of cellular senescence, it becomes evident that this field holds immense promise and challenges alike. As evident from our discussion, unraveling the intricacies of senescence will require interdisciplinary collaboration, innovative technologies, and a nuanced understanding of its biological underpinnings.

The rise of artificial intelligence and advanced technologies aligns perfectly with recent efforts by individuals [27,28] and broader communities to identify and localize senescent cells in vivo in different pathological contexts [29]. Notably, Cellular Senescence Network (SenNet), funded by the National Institutes of Health (NIH), among their other goals, is dedicated to characterizing senescent cells in human tissues to identify more precise markers in vivo, pushing the field toward faster therapies [30]. Clinical computational modeling pathology [31,32,33,34,35,36] has just begun to emerge, and its application on human-senescence-related histological maps will enable the extraction of actionable knowledge to generate prognostic models and accelerate clinical routine screening and patient stratification, facilitating the groundbreaking research we are looking for to fight aging and age-related diseases.

The connection between senescence and cancer is not at its end. Indeed, one of the most promising approaches for treating particularly high-aggressiveness tumors [37] has been adapted to protect the organism from senescent cells. The infusion of T cells engineered with urokinase-type plasminogen activator receptor (uPAR), a protein overexpressed on the surface of senescent cells, has been shown to improve fibrosis in mice, with long-lasting beneficial effects on their health [38,39]. While some uncertainties remain due to limited knowledge of potential side effects in healthy organs or in fragile patients, this senolytic-CAR T cell approach serves as proof of principle, demonstrating how harnessing the immune system to target senescent cells could open exciting new avenues in aging research.

The journey ahead is rich with possibilities.
